# Oxidative Stress and Nucleic Acid Oxidation in Patients with Chronic Kidney Disease

**DOI:** 10.1155/2013/301982

**Published:** 2013-08-24

**Authors:** Chih-Chien Sung, Yu-Chuan Hsu, Chun-Chi Chen, Yuh-Feng Lin, Chia-Chao Wu

**Affiliations:** ^1^Division of Nephrology, Department of Medicine, Tri-Service General Hospital, National Defense Medical Center, No. 325, Section 2, Cheng-Kung Road, Neihu, Taipei 114, Taiwan; ^2^Graduate Institute of Medical Science, National Defense Medical Center, Taipei 114, Taiwan; ^3^Division of Neurology, Department of Medicine, Taoyuan General Hospital, Ministry of Health and Welfare, Taoyuan 330, Taiwan; ^4^Division of Nephrology, Department of Medicine, Taipei Medical University-Shuang Ho Hospital, Ministry of Health and Welfare, New Taipei City 235, Taiwan; ^5^Graduate Institute of Clinical Medical, Taipei Medical University, Taipei 110, Taiwan; ^6^Graduate Institute of Microbiology and Immunology, National Defense Medical Center, Taipei 114, Taiwan

## Abstract

Patients with chronic kidney disease (CKD) have high cardiovascular mortality and morbidity and a high risk for developing malignancy. Excessive oxidative stress is thought to play a major role in elevating these risks by increasing oxidative nucleic acid damage. Oxidative stress results from an imbalance between reactive oxygen/nitrogen species (RONS) production and antioxidant defense mechanisms and can cause vascular and tissue injuries as well as nucleic acid damage in CKD patients. The increased production of RONS, impaired nonenzymatic or enzymatic antioxidant defense mechanisms, and other risk factors including gene polymorphisms, uremic toxins (indoxyl sulfate), deficiency of arylesterase/paraoxonase, hyperhomocysteinemia, dialysis-associated membrane bioincompatibility, and endotoxin in patients with CKD can inhibit normal cell function by damaging cell lipids, arachidonic acid derivatives, carbohydrates, proteins, amino acids, and nucleic acids. Several clinical biomarkers and techniques have been used to detect the antioxidant status and oxidative stress/oxidative nucleic acid damage associated with long-term complications such as inflammation, atherosclerosis, amyloidosis, and malignancy in CKD patients. Antioxidant therapies have been studied to reduce the oxidative stress and nucleic acid oxidation in patients with CKD, including alpha-tocopherol, N-acetylcysteine, ascorbic acid, glutathione, folic acid, bardoxolone methyl, angiotensin-converting enzyme inhibitor, and providing better dialysis strategies. This paper provides an overview of radical production, antioxidant defence, pathogenesis and biomarkers of oxidative stress in patients with CKD, and possible antioxidant therapies.

## 1. Introduction

Chronic kidney disease (CKD) and/or end-stage renal disease (ESRD) have a high incidence of cardiovascular disease and malignancy [1, 2]. Several factors contribute to both types of health consequences including immune system dysfunction, chronic inflammation and infection, reduced antioxidant levels, and accumulation of uremic toxins. The mortality rate is substantially higher in patients with CKD than in the general population, and increased oxidative stress has been observed in patients with CKD [3, 4].

Oxidative stress results from an imbalance between free radical production and insufficient endogenous antioxidant defense mechanisms and has been documented in uremic patients [5, 6]. Most free radicals in biological systems are aerobic metabolism-generated reactive oxygen species (ROS), but there are also derivatives of nitrogen (reactive nitrogen species, RNS) [7]. Increased concentration of malondialdehyde generated by lipid peroxidase [8] and impaired function of antioxidant systems because of low levels of superoxide dismutase and glutathione (GSH) peroxidase have been reported in hemodialysis (HD) patients [9]. These products can also induce chemical changes in many substances such as proteins, lipids, and nucleic acids. 

Oxidative nucleic acid damage is defined as the imbalance between the excess formation and insufficient removal of highly reactive molecules (ROS and RNS) in response to environmental or behavioral stress [10]. Oxidative stress can induce DNA or nucleic acid damage, such as base and sugar modifications [11], covalent crosslinks, and single- and double-stranded breaks [12]. The DNA bases, especially guanine (G), are particularly susceptible to oxidation, leading to oxidized guanine products. Nucleobase modifications most frequently involve 8-hydroxy-2′-deoxyguanosine (8-OH-dG), one of the most abundant oxidative products of nucleic acids [13]. In CKD patients, impaired function of the antioxidant systems and imbalance between free radicals and endogenous antioxidant forces may contribute to the accelerated development of oxidative nucleic acid damage, which may increase the risk of later cancer development [14]. The purpose of this review is to provide an overview of pathogenesis, biomarkers, and consequences of oxidative stress in patients with CKD and the possible antioxidant therapies to reduce oxidative stress and nucleic acid oxidation. 

## 2. Pathogenesis of Oxidative Stress and Nucleic Acid Oxidation in CKD

### 2.1. Increased Production of RONS in Patients with CKD (Figure 1)

#### 2.1.1. Generation of RONS by the Nicotinamide Adenine Dinucleotide Phosphate (NADPH) Oxidase Complex

The mitochondrial respiratory chain represents the most powerful cellular source of oxidants in the body. During respiration, in the mitochondrial electron transport chain, the electrons are passed individually to oxygen. Each oxygen molecule needs four electrons to be reduced completely; intermediate stages of reduction are formed during electron transport, thereby producing free radicals, which are atoms or molecules with one or more unpaired electrons that are capable of independent existence. Free radicals are particularly reactive molecules. Most free radicals in biological systems are derivatives of oxygen, but there are also derivatives of nitrogen (reactive nitrogen species, RNS) [7]. ROS and RNS are terms used collectively to describe highly reactive oxygen and nitrogen radicals, as well as nonradical derivatives.

Superoxide anion (O_2_^−^) is the major free radical generated *in vivo* by the reduction of molecular oxygen through the action of the NADPH oxidase enzyme complex. As soon as O_2_^−^ is formed, it is converted into hydrogen peroxide (H_2_O_2_). Excessive production of ROS by NADPH oxidase is commonly thought to be responsible for tissue injury associated with a range of chronic inflammatory diseases and has long been considered a unique property of phagocytic cells [15]. Both O_2_^−^ and H_2_O_2_ are precursors for the production of more powerful oxidants. O_2_^−^ has a high affinity for reacting with the free radical nitric oxide (NO), which rapidly produces the RNS peroxynitrite (ONOO^−^) [16], whereas H_2_O_2_ reacts with intracellular iron to form the hydroxyl radical (OH^−^) via the Haber-Weiss cycle. The resulting ONOO^−^ and OH^−^ can lead to extensive nitrosative and oxidative modifications to proteins [17], lipids [18], and nucleic acids [19].

#### 2.1.2. Generation of Chlorinated Oxidants by the Myeloperoxidase System

Myeloperoxidase, an abundant enzyme in macrophages and neutrophils, catalyzes the generation of the oxidant hypochlorous acid (OCl^−^) from H_2_O_2_ in the presence of Cl^−^. HOCl^−^ is a powerful compound capable of oxidizing chlorination of many molecules such as lipids, proteoglycans, amino acids, and other membranous or intracellular constituents [20]. Some studies have provided biochemical, experimental, and human clinical data supporting the important role of myeloperoxidase-catalyzed oxidation in atherosclerotic disease in individuals with uremia [21, 22].

In the progression of CKD, the redox balance is not in equilibrium and is tipped toward oxidation, resulting in the dysregulation of cellular processes and subsequent tissue injury. Several studies have reported on the overproduction of ROS in uremic patients treated by HD [23, 24], which occurs via priming of leukocytes to produce ROS and HD-induced activation of polymorphonuclear leukocytes.

### 2.2. Impairment of the Antioxidant System in Patients with CKD (Figure 2)

To prevent oxidative stress, detoxification of specific free radicals and other oxidants requires several intracellular and extracellular antioxidant systems, including both enzymatic and nonenzymatic systems.

#### 2.2.1. Antioxidant Nonenzymatic Systems

A thiol is a compound containing the functional group –SH (reduced) and can be oxidized via disulfide bond formation (oxidized). By reacting with almost all physiological oxidants, thiols function as key antioxidant buffers in the maintenance of the homeostatic intracellular and tissue reduction/oxidation (redox) state. Thiol-containing cysteine residues in proteins are sensitive to oxidation, and changes in enzymatic activity or binding characteristics caused by oxidation provide a mechanism for signal transduction [25, 26]. The major biological thiol/disulfide couples are GSH, thioredoxin, and other cysteine-containing proteins. GSH is a scavenger of H_2_O_2_, OH^−^, and chlorinated oxidants. Extracellular thiols also constitute an important component of antioxidant defense, particularly in plasma and interstitial fluids. Antioxidant defense is impaired when protective thiols are depleted in acute and chronic kidney injury, and oxidized thiols are toxic to the endothelium [27, 28].

Vitamin E (alpha-tocopherol) can protect cell membranes from lipid peroxidation and can interrupt the radical cascade by forming a low-reactivity vitamin that does not attack lipid substrates [14]. Vitamin E appears to be important in the protection against free-radical-induced oxidative damage by low-density lipoprotein (LDL) in biological membranes.

Vitamin C (ascorbic acid) is distributed widely in both intra- and extracellular fluids. Vitamin C acts as a potent water-soluble antioxidant in biological fluids by scavenging ROS (O_2_^−^ and OH^−^) and RNS species by forming semidehydroascorbic acid and may thereby prevent oxidative damage to important biological macromolecules [14]. Vitamin C deficiency in CKD patients on HD may be secondary to dietary restriction of fresh fruits and vegetables to avoid hyperkalemia and to loss of the vitamin when receiving dialysis.

Other inflammation proteins such as ferritin, transferrin, ceruloplasmin, and even albumin may also act as antioxidants by sequestrating transition metal ions involved in the formation of the most reactive oxyradicals.

#### 2.2.2. Antioxidant Enzymatic Systems

Superoxide dismutases (SODs) represent a major defense system against oxidative damage by enzymatically converting O_2_^−^ to H_2_O_2_. There are three types of SODs in mammalian tissues: copper-zinc-containing SOD (SOD1) localized in the cytosol, manganese-containing SOD (SOD2) localized in the mitochondrial matrix, and extracellular SOD (SOD3). SOD1, SOD2, and SOD3 are each highly expressed in the normal kidney, predominantly in the renal tubules, compared with other organs [25]. A recent study suggests that SOD1 is a major antioxidant enzyme in the regulation of oxidative stress during progressive renal injury [29].

Catalase, which is responsible for the reduction of H_2_O_2_ to water, is expressed in most cells, organs, and tissues and at high concentrations in the liver and erythrocytes. It may also be a key enzyme in antioxidant defense in the kidney during injury. A recent study reported that the loss of catalase-buffering capacity leads to an increase in oxidative products and more severe renal fibrosis, resulting in progressive kidney disease in catalase-deficient mice [30].

Other antioxidants, including peroxiredoxin, thioredoxin reductase, and GSH peroxidase (GPx), also represent an important group of predominantly intracellular enzymes that reduce and inactivate H_2_O_2_ and other organic peroxides to water and oxygen. Five isoforms of GPx have been identified; two are present in human blood, GPx 1 in red blood cells [31], and GPx 3, which is produced by the kidney, in plasma [32].

The thioredoxin system comprises thioredoxin, thioredoxin reductase, and peroxiredoxin, which represent highly abundant proteins that are distributed through the cytoplasm, mitochondria, and other cell compartments [33]. Selenium-containing GPx reduces all organic lipid peroxides and requires GSH as a hydrogen donor [34]. Profound deficiencies in the activity of the GSH system and in selenium have been reported in HD patients. GPx activity is altered significantly in the early stages of CKD, decreases with the progression of uremia, and decreases markedly in HD patients [35]. The potential deleterious effect of HD-induced ROS overproduction is augmented by the impairment of antioxidant defense mechanisms associated with uremia; the main disturbance concerns the GSH/GPx/selenium complex because selenium concentration decreases significantly in uremic patients [36].

### 2.3. *hOGG1* Gene and Other Gene Polymorphisms

Genetic background is known to be involved in the control of damaged DNA repair. Genetic polymorphisms in DNA repair genes may affect DNA repair capacity, resulting in DNA damage accumulation. The base excision repair (BER) pathway, containing *hOGG1*,* MTH1,* and *MUTYH*, is a major protector from oxidative DNA damage in humans. A C → G polymorphism at position 1245 in exon 7 of the *hOGG1* gene is associated with the substitution of cysteine for serine at codon 326 (Ser326Cys) and is a determinant of genomic damage in leukocytes [37, 38]. A population with decreased enzyme activity of the *hOGG1* gene would be at risk of accumulating 8-OH-dG in nuclear DNA because of incomplete repair of oxidatively damaged DNA. In one study of patients undergoing chronic HD, leukocyte 8-OH-dG level was increased further among patients with the 1245 GG genotype compared with patients with the 1245 CG or CC genotype [37]. Another recent report in Turkey showed that *XRCC1* Arg399Gln polymorphism may increase the risk for the development of ESRD [39]. In a Chinese population study, the polymorphisms in BER system, including *MUTYH* c.972GG (rs3219489) and *AluYb8MUTYH *(rs10527342), increased the risk for ESRD development, especially their combined effect with *OGG1*c.977GG. Therefore, those homozygous or heterozygous BER polymorphisms might be candidate genetic factors for ESRD development [40]. Oxidative DNA damage among chronic HD patients is influenced by a combination of the overproduction of ROS, impaired antioxidant defense mechanisms, and genetic influence.

Single-nucleotide polymorphisms (SNPs) of antioxidant enzymes including SOD2, GPx, and catalase may contribute to diseases associated with oxidative stress [41]. These diseases include cancer, diabetes, Alzheimer's disease and other neurodegenerative diseases, cardiovascular disease, and CKD [42]. The antioxidant enzyme SNPs associated most frequently with disease in humans are SOD2 SNP Ala16Val, GPx1 SNP Pro197Leu, and catalase SNP C-262T. Most evidence supports associations between the SOD2 SNP Ala16Val genotype and diseases such as breast, prostate, and lung cancers, diabetes, and cardiovascular disease, whereas the GPx1 SNP Pro197Leu and catalase SNP C-262T SNP genotypes are associated with breast cancer [43]. It has been reported that CKD patients with the SOD Ala/Val and Val/Val genotypes have a significantly greater decline in estimated glomerular filtration rate (eGFR) compared with patients with the Ala/Ala genotype. The amino acid change from Ala to Val affects the structure of SOD, changing the alpha helix structure to a beta sheet. Therefore, the SOD genotype may be useful for identifying CKD patients at risk of more rapid CKD progression [41].

GSH S-transferase M1 (GST M1) is a member of the GST family of proteins, which protects cellular DNA against oxidative damage. Patients undergoing maintenance HD who lack GST M1 activity (a particular GST M1 polymorphism) are more vulnerable to oxidative stress and are at greater risk of death compared with those who possess GST M1 activity [44].

### 2.4. Other Factors That Can Induce Oxidative Stress in CKD

#### 2.4.1. Uremic Toxins

In CKD patients, progressive deterioration of renal function can lead to accumulation of uremic toxins, which can induce oxidative stress [52]. Indoxyl sulfate (IS), a uremic toxin, is an organic anion that is normally excreted into urine and exists at high concentrations in the serum of patients with progressive CKD [53]. A high concentration of IS is considered a risk factor for cardiovascular disease in CKD patients and accelerates the progression of CKD. It has been reported that IS upregulates the expression of intercellular adhesion molecule-1 (ICAM-1) and monocyte chemotactic protein-1 (MCP-1) by ROS-induced activation of NADPH oxidase and nuclear factor-*κ*B (NF-*κ*B) in vascular endothelial cells. Thus, IS may play an important role in the development of cardiovascular disease in CKD patients by increasing the endothelial expression of ICAM-1 and MCP-1 [54].

#### 2.4.2. Homocysteine (Hcy)

The sulfur-containing amino acid Hcy is a prominent uremic toxin and is a normal product in the metabolism of the essential amino acid methionine. Hcy is an intermediary amino acid formed endogenously by the conversion of methionine to cysteine. In healthy individuals, Hcy is remethylated to methionine (predominantly in the kidney) or can be metabolized by the transsulfuration pathway to cysteine. The vast majority of patients with end-stage renal disease (ESRD) have high plasma Hcy levels, but the reasons involve different mechanisms, which may include reduced renal and nonrenal organic clearance [55]. Vitamin deficiency, mainly of folic acid and vitamin B_12_ (cobalamin), is considered a major contributor to the hyperhomocysteinemia found in patients with CKD. High blood Hcy levels increase oxidative stress because Hcy is susceptible to autooxidation, with secondary generation of ROS, and Hcy can inhibit the activity of the antioxidant enzymes GPx and SOD [56]. Hyperhomocysteinemia in patients with CKD is considered a risk factor for malignancy because the DNA of these patients is hypomethylated [57, 58].

#### 2.4.3. Arylesterase/Paraoxonase

Human arylesterase (PON1), member of the paraoxonase family of enzymes, hydrolyses organophosphate compounds and has a protective effect against lipoprotein oxidation in CKD. Most importantly, PON1 displays Hcy-thiolactonase activity and poses antiatherogenic properties. PON1 is diminished in CKD patients when compared to healthy controls and might be a sensitive marker of antioxidant status [59, 60].

#### 2.4.4. HD-Induced Oxidative Stress

Uremic toxins, dialyzer interactions, and dialysate contaminants have been suggested as the three major causes of oxidative stress in HD patients. The dialysis membranes seem to play a central role in the increased production of ROS in these patients [61, 62]. Our previous study [62] to evaluate the influence of two different dialysis membranes, polysulfone compared with regenerated cellulose (RC), on oxidative stress during HD found that HD with RC membranes resulted in a significantly increased production of oxidants during a single HD session, whereas dialysis with a polysulfone dialyzer had a milder effect. HD may induce repetitive bouts of oxidative stress, which may trigger the generation of ROS, primarily through membrane bioincompatibility and endotoxin (LPS) challenge [63]. Hemoincompatibility of the dialysis system plays a critical role in the production of ROS. LPS in the dialysate may indirectly trigger ROS production by activating polymorphonuclear leukocytes. Moreover, because HD can also reduce the levels of oxidized protein thiols, HD modalities using highly permeable membranes can cause solute loss, including loss of hydrophilic nonenzymatic antioxidants [64]. However, HD treatment can also improve oxidative status and reverse the increased levels of oxygen radical production by neutrophils in the blood of patients with ESRD [64].

## 3. Biomarkers of Oxidative Stress and Nucleic Acid Oxidation in CKD

In 1994, Maggi et al. first reported that oxidative stress may contribute to atherosclerosis in uremic patients by measuring changes in lipid peroxidation [65]. Uremic oxidative stress can be characterized biochemically as a state of accumulation of reactive aldehyde and oxidized thiol groups, with a concomitant depletion of reduced thiol antioxidant groups. Numerous methods to estimate the degree of oxidative stress have been used, ranging from techniques with low dynamic ranges (such as immunostaining) to powerful analytical assays such as liquid chromatography or gas chromatography coupled with mass spectrometry. The most commonly used biomarkers in human and experimental models are listed in Table 1. Excess prooxidants or free radicals can oxidize macromolecules such as lipids, proteins, carbohydrates, and nucleic acids, causing DNA, cellular, and tissue injury. A promising, more complete approach may involve the simultaneous use of multiple biomarkers from both the oxidant-generating and antioxidant pathways to assess oxidative stress [66]. One recent systemic review reveals that several biomarkers emerged as well-suited indicators of oxidative stress and antioxidant status in patients with CKD including malondialdehyde, F2-isoprostanes, lipid hydroperoxides, asymmetric dimethylarginine (ADMA), protein carbonyls, advanced oxidation protein products (AOPPs), 8-oxo-7,8-dihydro-2′-deoxyguanosine (8-oxo-dG), and glutathione-related activity [60].

### 3.1. Biomarkers of Lipid Peroxidation, Protein Oxidation, and Protein Carbonylation

#### 3.1.1. Lipid Peroxidation

Peroxynitrite is generated from the reaction of NO with ROS and has several unfavorable vascular actions. Although the availability of NO is reduced, the increased production of advanced glycation end products (AGEs) adds to the atherogenic potential of renal insufficiency. Measurements of lipid hydroperoxides (oxidized low-density lipoprotein (LDL) and HOC1-modified LDL) and nitrated amino acids serve as excellent markers of cell and tissue oxidative stress because of their relative stability compared with the direct measurement of more transient free radicals. ROS can react with double bonds of polyunsaturated fatty acids (PUFAs) to yield lipid hydroperoxides. Malondialdehyde is the secondary oxidation product of peroxidized PUFAs and has been shown to have mutagenic and cytotoxic effects and possibly to be involved in the pathogenesis of several human diseases, including atherosclerosis, neurodegenerative diseases, and cancer [67]. Lipid peroxidation products, namely, malondialdehyde [68], 4-hydroxynonenal (HNE) [69], advanced lipoxidation end products (ALE) [70], hydroxyoctadecadienoic acid (HODE) [71], F_2_-isoprostanes [72] (enzymatically produced by free-radical-catalyzed peroxidation of arachidonoyl lipids), and isolevuglandins, have been reported to be elevated in HD patients [73]. The thiobarbituric-acid-reactive substances (TBARS) and lipoperoxides have been reported to be increased in patients after one year of HD [74, 75]. Cholesteryl esters (CE) in the hydrophobic core of LDL particles are oxidized to hydroperoxides. CE are sensitive markers of lipid damage and have been used to examine oxidatively damaged tissues [60].

#### 3.1.2. Biomarkers of Protein Oxidation Damage

In contrast to lipids, reaction products of protein with various oxidants can accumulate, and the subsequent reactants may have toxic activities. Evaluation of oxidatively modified proteins may be useful in assessing oxidative stress status. Protein oxidation markers had not been documented thoroughly in HD patients until AOPPs were identified in the plasma of uremic patients [76]. AOPPs can be detected in HD patients and in CKD patients not yet on dialysis. Oxidation of plasma thiol groups is quantitatively the major manifestation of protein oxidation.

#### 3.1.3. Biomarkers of Protein Carbonylation and Amino Acid Damage

Oxidative stress may contribute to the progression of renal disease through the generation of AGEs. AGEs are formed nonenzymatically by the reaction of carbonyl compounds with a free amino group from proteins, lipids, or amino acids and have been identified as markers of oxidative stress in uremic patients. The components of oxidative stress that increase production of AGEs by increasing the formation of carbonyl groups are termed carbonyl stress compounds [77, 78]. AGEs accumulate during aging and in the course of many degenerative diseases and can be removed by the kidney. AGEs have genotoxic effects and can react with DNA in a similar way to their reaction with proteins, resulting in the formation of DNA-bound AGEs [79].

The aromatic amino acids are very susceptible to oxidation by various ROS. For example, OH^−^-radical-mediated oxidation of tyrosine residues gives rise to dityrosine; reaction with RNS leads to the formation of 3-nitrotyrosine; reaction with HOCl leads to the generation of 3-chlorotyrosine; lysine residues are oxidized to carboxymethyl lysine; and cysteine residues are also oxidized to cysteine/cystine and Hcy/homocystine. 3-Nitrotyrosine is an oxidation byproduct that accumulates in fibrotic kidneys and in the serum of patients with CKD, suggesting that oxidative stress increases during the progression of kidney disease. 

ADMA, an analogue of L-arginine, is a naturally occurring product of metabolism found in human circulation. ADMA can uncouple endothelial NO synthase, leading to the loss of NO and an increase in superoxide production in the vascular endothelium [60]. In progression of CKD, elevated levels of ADMA can inhibit NO synthesis and therefore impair endothelial function and thus promote atherosclerosis.

### 3.2. Biomarkers of Oxidative Nucleic Acid Products in Patients with CKD

The OH^−^ attacks DNA strands when it is produced adjacent to cellular and mitochondrial DNA (mtDNA), causing the addition of DNA bases containing new radicals, which lead to the generation of a variety of oxidation products [80]. The interaction of OH^−^ with the nucleobases of the DNA strand, such as guanine, leads to the formation of C8-hydroxyguanine or its nucleoside form 8-OH-dG. Initially, the reaction of OH^−^ addition leads to the generation of radical adducts, after which one electron abstraction leads to the formation of 8-OH-dG. 8-OH-dG undergoes keto-enol tautomerism, which favors the oxidized product 8-oxo-dG. In nuclear DNA and mtDNA, 8-OH-dG and 8-oxo-dG are the predominant forms of free-radical-induced oxidative lesions and are therefore used widely as biomarkers for oxidative stress and carcinogenesis. The biomarkers 8-OH-dG and 8-oxo-dG are pivotal markers for measuring the effects of endogenous oxidative damage on DNA and are factors involved in the initiation and promotion of carcinogenesis [81]. Usually, 8-OH-dG is measured as an index of oxidative DNA damage. Although the other nucleobases of DNA react with OH^−^ in a similar manner, lesions associated with 8-oxo-dG are the most abundant DNA lesions because they form easily; 8-oxo-dG is promutagenic and is therefore a potential biomarker of carcinogenesis [82]. 8-OH-dG level can be measured in animal organs and in human samples such as in the urine, organs, and leukocyte DNA and serves as a biomarker of oxidative stress, aging, and carcinogenesis [83, 84].

8-OH-dG is one of the most abundant oxidative DNA products among the base modifications elicited by ROS and may provide a new marker for the assessment of oxidative DNA damage in ROS-mediated diseases [13, 85–87]. The 8-OH-dG level in leukocytes is significantly higher in HD patients compared with nondialyzed patients with advanced renal failure and with healthy subjects [88]. Markedly elevated 8-OH-dG levels have been reported in patients on peritoneal dialysis therapy [89].

### 3.3. Techniques to Detect DNA Damage

Both before starting HD and during HD, peripheral blood leukocytes of CKD patients exhibit elevated genomic or nucleic acid damage compared with healthy controls. These changes have been demonstrated by analyzing the 8-OH-dG content in leukocyte DNA, micronuclei frequency, single-cell gel electrophoresis (comet assay), sister chromatid exchange or mitochondrial DNA deletions to detect the oxidatively damaged DNA, and nucleic acid damage. DNA and nucleic acid damage are measured in peripheral blood lymphocytes (PBL) as biomarkers of the early effects of genotoxic carcinogens in occupational and environmental settings.

#### 3.3.1. 8-OH-dG

It is generally accepted that oxidatively damaged DNA can be repaired and that the repair products are released into the bloodstream and consequently appear in the urine without further metabolism [90]. The first report of an analysis of 8-OH-dG as a major oxidation product of DNA in the urine from experimental animals and humans was published in 1989 [91]. Studies have shown that urinary 8-OH-dG level is a good biomarker for risk assessment of various cancers and degenerative diseases. Various analytical techniques have been developed to measure oxidatively damaged DNA in the urine, including high-performance liquid chromatography with electrochemical detection, gas-chromatography-mass spectrometry, enzyme-linked immunosorbent assay (ELISA), and liquid-chromatography-tandem mass spectrometry (LC-MS/MS) [92]. The first two methods have been established for >10 years, although they are labor intensive and have inadequate specificity or require chemical derivatization. A commercial ELISA kit is the most convenient method and is used frequently, although it often overestimates urinary 8-OH-dG concentration compared with chromatographic procedures. LC-MS/MS is a relatively new and powerful technology that can overcome the sensitivity and selectivity issues in the analysis of DNA adducts [93].

#### 3.3.2. Micronuclei Frequency

Micronuclei are chromatin-containing structures surrounded by a membrane that are formed during mitosis [94]. The formation of micronuclei in dividing cells is the result of chromosome breakage caused by unrepaired or misrepaired DNA lesions, or chromosome malsegregation caused by mitotic malfunction. Micronuclei are sensitive *in vivo* and *in vitro* indicators of exogenous and endogenous genetic damage. Micronuclei and other nuclear anomalies such as nucleoplasmic bridges and nuclear buds are biomarkers of genotoxic events and manifestations of chromosomal instability that are often seen in cancer. The frequency of micronuclei in PBL is used extensively in molecular epidemiology and cytogenetics to evaluate the presence and the extent of chromosomal damage in human populations exposed to genotoxic agents [95]. Elevated rates of sister chromatid exchange and abnormal chromosomes have also been observed [96].

#### 3.3.3. Single-Cell Gel Electrophoresis (Also Known as a Comet Assay) in PBL

The comet assay or single-cell gel electrophoresis is a simple and sensitive technique for detecting DNA damage at the level of the individual eukaryotic cell; this assay was first developed by Östling and Johansson in 1984 and later modified by Singh et al. in 1988 [97]. The comet assay measures DNA strand breaks, alkali labile sites, and relaxed chromatin in individual cells [97, 98]. In this assay, the cells are embedded in agar and exposed to an electrical field. Most of the genetic material from cells with damaged DNA migrates faster than does the material from cells with intact nuclear DNA. DNA migration is measured using computer assistance [99].

#### 3.3.4. Mitochondrial DNA (mtDNA) Deletions

Mitochondria are a major intracellular source of ROS and free radicals. Because it lacks protective histones and has a low efficacy of DNA repair, mtDNA seems to be particularly sensitive to ROS [100]. Compared with nuclear DNA, human mtDNA is much more susceptible to damage induced by mutagens or carcinogens, such as free radicals, caused by the lack of proofreading and poor DNA repair during mtDNA replication. mtDNA deletions have been analyzed in hair follicles of patients with end-stage renal failure [101].

### 3.4. Biomarkers of Antioxidant Status in Patients with CKD

Antioxidant systems can stabilize free radicals, consequently reducing the oxidative stress. Enzymatic antioxidants are the most important defense against radical-induced damage. Therefore, recent researchers use different techniques to evaluate the activity of antioxidant status including oxidative stress index (OSI: ratio of total antioxidant capacity/total oxidant status), glutathione activity (GSH), superoxide dismutase (SOD), catalase, thioredoxin, and PON1/paraoxonase [60]. They also use different terms to express antioxidant status including total antioxidant efficiency, effectiveness, action, power, parameter, potential, potency, and activity. GSH is diminished in patients with CKD, even in the absence of dialysis. The ratios of GSH/GSSH and GSH-related makers including GSH-peroxidase, GSH-reductase, and GSH-S-transferase are also used to assess antioxidant status. Although SOD is a major antioxidant enzyme in the regulation of oxidative stress in CKD, the relationship between superoxide and SOD activity in CKD remains uncertain. Studies examining catalase activity in CKD are contradictory, but catalase might reflect antioxidant status in diabetes, rather than CKD. Besides, thioredoxin and PON1/paraoxonase are considered as the novel makers of antioxidant status in kidney disease [60]. 

## 4. Consequences of Oxidative Stress and Oxidative Nucleic Acid Damage in Patients with CKD

Oxidative stress, which can act synergistically with inflammation, is thought to be involved in the development of long-term complications such as inflammation, atherosclerosis, amyloidosis, and malignancy in CKD patients. Several lines of evidence indicate that nucleic acid damage associated with oxidative stress increases in CKD and HD patients. Stoyanova et al. [102] reported recently that oxidative nucleic acid damage was higher in HD (*n* = 77) than in CKD patients (*n* = 176). Another study reported that patients with CKD exhibit upregulation of a number of genes involved in the oxidative phosphorylation system, suggesting that an impaired mitochondrial respiratory system contributes to increased oxidative stress [103]. Using comet studies, Stopper et al. showed significant increases in the percentage of DNA in the comet “tail” (indicating DNA damage) in 23 CKD patients compared with 21 healthy subjects; nucleic acid damage was higher in patients with high creatinine levels [104]. Reduced nucleic acid repair capacity in predialysis patients [105] also increases oxidative nucleic damage.

### 4.1. Inflammatory Response and Apoptosis

Oxidative stress may play multiple roles in the inflammatory response by cytokine-related ROS release and by regulation of transcription factors. First, oxidant generation is amplified by proinflammatory cytokines, especially interleukin-6 (IL-6), IL-1*β*, and tumor necrosis factor-*α* (TNF-*α*), produced by monocytes and the acute-phase reactant C-reactive protein released in response to ROS [14]. Second, O_2_^−^ may activate NF-*κ*B and activator protein-1 (AP-1), leading to the expression of cytokines, which may in turn stimulate overproduction of ROS by the NADPH oxidase complex [106]. Third, activated NADPH oxidase (NOX) is now recognized as an important modulator of a specific intracellular signal-transduction pathway by activating redox-sensitive kinases. NOX4-mediated generation of O_2_^−^ leads to specific ERK1/2 and JNK activation. Angiotensin II and TNF-*α* can also lead to NOX2-mediated O_2_^−^ generation via JNK activation [107]. In addition, receptor binding of TGF increases production of ROS via the NOX2 and activated mitogen-activated protein kinase pathways, and subsequent binding of Smad proteins to the plasminogen-activator inhibitor-1 (PAI-1) promoter activates gene transcription. The addition of the thiol-containing antioxidant GSH blocks the TGF-*β*-stimulated transcription of PAI-1 [108, 109], and attenuation of the NOX-mediated redox signal leads to dysregulation of the acute inflammatory process.

Apoptosis is initiated by a variety of stimuli, including DNA damage, toxins, oxidant stress, and cytokines (especially TNF-*α*) [110]. Galli et al. have shown that the apoptotic rate is increased in chronic HD patients and that this increase is associated with oxidative stress [111]. C-reactive protein levels and Fas and TNF-R2 expression are higher in lymphocytes and monocytes from chronic HD patients compared with normal controls [112]. Apoptosis can be initiated by death-signal-inducing receptors, of which Fas (CD95) is the best known. In our previous study, we reported increased immunofluorescence of the apoptosis marker for Fas, CD95, in monocytes from uremic patients [110].

### 4.2. Atherosclerosis

Atherosclerosis, a recognized inflammatory disease, is strongly related to oxidative stress. Several qualitative changes in LDL oxidizability have been shown in dialysis patients, including increased carbamylation, AGE transformation, and oxidation, which all favor the development of atherosclerosis [113]. LDL-mediated oxidation may occur through generation of O_2_^−^ by macrophage 15-lipoxygenase and NADPH oxidase [114] or by endothelial NO synthase (eNOS) [115]. In the initial stages of atherosclerosis, eNOS activity may increase, supporting the concept of a role of oxidative stress in the early atherosclerosis of ESRD patients. The normal equilibrium between NO formation and ROS appears to be disturbed in the process of atherosclerotic plaque formation [116].

### 4.3. Amyloidosis

*β*_2_-Microglobulin is a major constituent of amyloid fibrils in HD-associated amyloidosis. *β*_2_-Microglobulin appears to be a good candidate for oxidative attack after the appearance of AGEs in amyloid deposits in long-term HD patients [117]. It has been reported that *β*_2_-microglobulin can be fragmented and polymerized following exposure to O_2_^−^ and OH^−^ generated by radiolysis [118].

### 4.4. DNA-Damage-Associated Malignancy

ESRD is associated with excessive morbidity and mortality because of cardiovascular disease resulting from inflammation and atherosclerosis and with increased occurrence of various types of cancer. The frequency of cancer is higher in patients with ESRD than in the general population [1, 2]. Oxidative stress and inflammation have been demonstrated to be of high pathogenetic relevance to cancer, especially because of the contribution of genomic damage. Oxidative damage to DNA may cause mutations of oncogenes and tumor-suppressor genes and may represent one mechanism underlying carcinogenesis [119–121]. AGEs are thought to be genotoxic. The most abundant of these lesions, 8-OH-dG, is also the most mutagenic and results in G → T transversion, which is found frequently in tumor-relevant genes. Other oxidative modifications of base and sugar residues in DNA occur frequently, but they have been studied less and their biological significance is unclear [120]. Stoyanova et al. [102] reported that 25% of patients with CKD have cancers, independent of whether they are undergoing HD treatment. Thus, DNA oxidative damage has also been identified as a useful index of oxidative stress and a possible indicator of cancer risk.

## 5. Antioxidant Therapies to Reduce Oxidative Stress and Nucleic Acid Oxidation in CKD and HD Patients (Table 2)

Antioxidant therapy may be beneficial for uremic patients with increased oxidative stress because an increase in oxidative stress contributes to uremic cardiovascular toxicity. Several products of oxidative metabolism have been reported to accumulate in the damaged kidney.

### 5.1. Vitamin E (Alpha-Tocopherol)

Some small studies of uremic patients have shown that the chain-breaking antioxidant alpha-tocopherol (vitamin E) has a biochemical efficacy in beneficially altering the biomarkers of oxidative stress and in increasing erythropoiesis or reducing the required dose of erythropoietin [122]. High-dose vitamin E supplementation has been associated with inhibition of proatherogenic events such as monocyte O_2_^−^ release, release of IL-1*β* from activated monocytes, lipid oxidation, platelet aggregation, *in vivo* smooth muscle cell proliferation, and monocyte adhesion to the endothelium. Vitamin E may help stabilize atherosclerotic plaque [123, 124]. The Secondary Prevention with Antioxidants of Cardiovascular Disease in End-Stage Renal Disease (SPACE) study reported a clinically and statistically significant reduction in the number of myocardial infarctions and other cardiovascular events in an alpha-tocopherol-treated group of HD patients compared with a group given placebo [45]. However, vitamin E failed to beneficially affect cardiovascular outcomes in the Heart Outcomes Prevention Evaluation (HOPE) study of 993 people with mild-to-moderate renal insufficiency treated with natural-source vitamin E (400 IU/day RRR-alpha-tocopherol acetate) or placebo [46]. Several reasons can explain the different cardiovascular outcomes between the SPACE and HOPE studies. First, the population enrolled in the SPACE trial was at higher cardiovascular risk. Second, participants in the SPACE study were treated with a higher dose of vitamin E than were those in the HOPE study. Third, most participants in the SPACE trial (43.3% of the vitamin E group) consumed vitamin C. Finally, the SPACE study included a smaller sample (196 HD patients) compared with other trials, which resulted in large confidence intervals based on a broad composite end point.

### 5.2. N-Acetylcysteine

Acetylcysteine, a thiol-containing antioxidant, has been used successfully to ameliorate the toxic effects of ischemia-reperfusion syndromes of the heart, kidney, lung, and liver [125, 126]. The activity of acetylcysteine is related to its action as a free radical scavenger or as a reactive sulfhydryl compound that increases the reducing capacity of the cell and may improve coronary and peripheral vascular function [127]. In patients undergoing HD (134 patients), treatment with oral acetylcysteine (600 mg two times/day) reduced the number of composite cardiovascular end points: cardiac events were reduced by 30%, and ischemic stroke was reduced by 36% [47]. Hsu et al. demonstrated that treatment of HD patients with acetylcysteine significantly improved anemia and decreased the plasma levels of 8-isoprostane and oxidized LDL [128]. Other studies have also shown that the use of N-acetylcysteine can reduce plasma Hcy concentration and improve endothelial function in HD patients, suggesting a direct mechanism for the improvement in endothelial function with antioxidant therapy [28, 129].

### 5.3. Ascorbic Acid (Vitamin C)

Vitamin C is depleted in fluids under *in vivo* conditions of oxidative stress such as smoking and inflammation associated with rheumatoid arthritis. Vitamin C also decreases lymphocyte 8-OH-dG content, markedly reduces intracellular ROS production by lymphocytes, and upregulates *hOGG1* mRNA expression in lymphocytes [48].

### 5.4. Angiotensin-Converting Enzyme Inhibitors

There is some evidence that other medications, including angiotensin-converting enzyme inhibitors or angiotensin II receptor antagonists, might also exert antioxidant effects [95, 130–132]. Both AGEs and angiotensin II are known to upregulate NADPH oxidase, causing ROS formation and subsequent activation of NF-*κ*B. The newly formed angiotensin II might amplify the AGE-induced DNA damage by increasing oxidative stress [133, 134]. The beneficial action of the angiotensin 1 receptor blockade in preventing DNA damage *in vivo* was confirmed in peripheral lymphocytes of rats with chronic renal failure (4/6 nephrectomy) [135]. Losartan has been found to decrease OSI and increase plasma levels of thiol groups in ESRD patients undergoing hemodialysis [49].

### 5.5. GSH and a Cysteine Prodrug

Oral administration of l-2-oxothiazolidine-4-carboxylate, a cysteine prodrug, has been shown to significantly elevate the whole-blood GSH level in chronic peritoneal dialysis patients [136].

### 5.6. Folic Acid and Vitamin B_12_

Folic acid, which is required to metabolize Hcy to methionine, lowers plasma Hcy levels in patients with CKD. Adding vitamin B_12_ reduces plasma Hcy level further. Routine supplementations with folic acid and other antioxidant vitamins should be considered in HD patients to lower Hcy levels; although these supplements may not normalize the Hcy level, lowering the Hcy level may be beneficial by reducing the cardiovascular risk in this group [50, 137, 138]. Folic acid can lower the frequency of micronuclei in PBL *in vitro* [139]; micronuclei frequency was lowered by 6-month folic acid supplementation [140]. Vitamin B_12_ and folic acid are also important modulators of the micronuclei frequency in PBL of healthy individuals [141, 142].

### 5.7. Bardoxolone Methyl

Nuclear-factor-erythroid-2-related factor 2 (Nrf2) has a central role in the basal activity and coordinated induction of over 250 genes encoding antioxidant enzymes and related proteins, such as SOD, catalase, NADPH:quinine oxidoreductase-1, glutathione S-transferase, glutathione peroxidase, and thioredoxin. Nrf2 is held in the cytoplasm as an inactive complex bound to Keap 1 (Kelch-like ECH-associated protein 1), a repressor molecule that facilitates Nrf2 ubiquitination [143]. Bardoxolone methyl, an antioxidant modulator of inflammation, activates the Keap1-Nrf2 pathway, which plays an important role in maintaining kidney function and structure. In a recent study, treatment with bardoxolone methyl at a target dose of 25, 75, or 150 mg once daily for 52 weeks led to sustained, significant improvements in the eGFR rate in patients receiving standard medical care for CKD and type 2 diabetes [51].

Bardoxolone methyl interacts with cysteine residues on Keap1, allowing Nrf2 translocation to the nucleus and subsequent upregulation of a multitude of cytoprotective genes. The structure and activity profile of bardoxolone methyl resemble those of the cyclopentenone prostaglandins, endogenous Nrf2 activators that promote the resolution of inflammation. Bardoxolone methyl has anti-inflammatory effects by inhibiting the proinflammatory NF-*κ*B pathway [51, 144–148]. Bardoxolone methyl appears to be an attractive therapeutic candidate for further study in patients with CKD.

### 5.8. New Dialytic Techniques

It has been reported that a daily HD (6 times/week) regimen can effectively lower the mean levels of glycation-related substances compared with standard HD (3 times/week). Therefore, daily HD can provide better control of AGEs produced in ESRD [149].

## 6. Conclusion

Patients with CKD have high morbidity and mortality compared with healthy people. Excessive oxidative stress resulting from an imbalance between RONS and antioxidative mechanisms has been documented in these patients and may reflect genetic influences. Different strategies to reduce oxidative nucleic acid damage in these patients and the development of biomarkers have provided evidence of the efficacy of antioxidants and new dialytic techniques in preventing oxidative stress in CKD patients, but further work is needed to evaluate these antioxidant therapies fully.

## Figures and Tables

**Figure 1:**
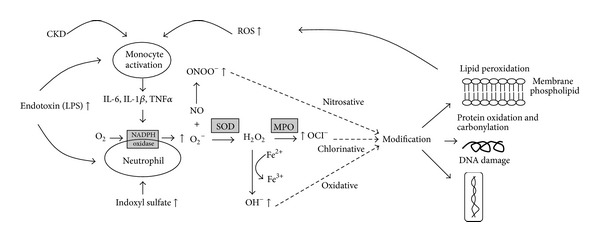
Synthesis of reactive oxygen species (ROS) in patients with chronic kidney disease (CKD). Excessive reactive ROS including ONOO^−^, OH^−^, and OCl^−^ are generated from oxygen through several main enzymes (NADPH oxidase, superoxide dismutase (SOD), and myeloperoxidase (MPO)). Several factors can also increase ROS generation, including cytokines (IL-8, IL-1*β*, and TNF-*α*) released from activated monocytes, uremic toxin (indoxyl sulfate), and endotoxin (LPS) from the HC procedure. The resulting excessive ROS can lead to nitrosative (ONOO^−^), chlorinative (OCl^−^), and oxidative (OH^−^) modifications to lipids, proteins, and DNA.

**Figure 2:**
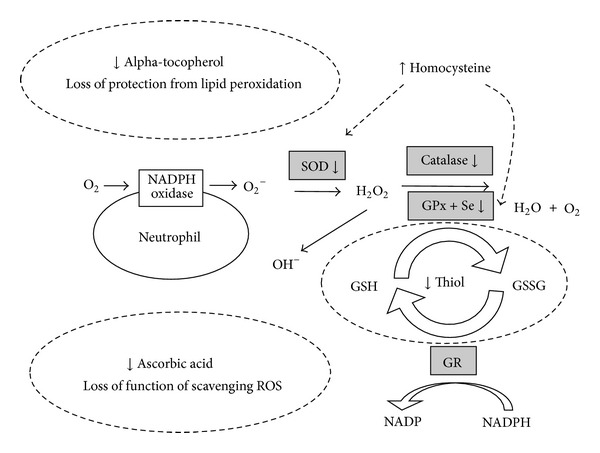
Impairment of antioxidant system in patients with CKD. Antioxidant systems, including nonenzymatic systems (thiol, alpha-tocopherol, and ascorbic acid) and enzymatic systems (superoxide dismutase (SOD), catalase, and glutathione peroxidase (GPx)), are impaired or deficient in patients with CKD. Hyperhomocysteinemia can lead to inhibition of the activity of the antioxidant enzymes SOD and GPx. GR: glutathione reductase; GSH: glutathione; GSSG: glutathione disulfide; Se: selenium.

**Table 1:** Biomarkers of oxidative stress and antioxidant status.

Lipid peroxidation	Oxidized low-density lipoprotein (LDL), HOC1-modified LDL, malondialdehyde (MDA), 4-hydroxynonenal (HNE), hydroxyoctadecadienoic acid (HODE), thiobarbituric-acid-reactive substances (TBARS), advanced lipoxidation end products (ALE), cholesteryl esters

Arachidonic-acid-derived oxidation	Isofurans, F_2_-isoprostane, isolevuglandins

Protein oxidation	Advanced oxidation protein products (AOPPs), protein thiols oxidation

Protein carbonylation	Advanced glycation end products (AGEs)

Amino acid oxidation	3-Nitrotyrosine, 3-chlorotyrosine, dityrosine, carboxymethyl lysine, cysteine/cystine, homocysteine/homocystine

Nucleic acid oxidation	8-Oxo-7,8-dihydro-2′-deoxyguanosine (8-oxo-dG), 8-hydroxy-2′-deoxyguanosine (8-OH-dG)

Antioxidant status	Oxidative stress index (OSI: ratio of total antioxidant capacity/total oxidant status), glutathione activity, superoxide dismutase, catalase, thioredoxin, arylesterase/paraoxonase

**Table 2:** Antioxidant therapies to reduce oxidative stress in CKD and HD patients.

Study	Intervention	Subjects	Effect
*Vitamin E *			
Boaz et al. (2000) [45] SPACE study	High-dose alpha-tocopherol (800 IU once daily) or placebo	196 HD patients with preexisting cardiovascular disease followed for a median of 519 days	(1) Significant reduction in myocardial infarctions and other cardiovascular events (2) No significant difference in overall survival
Mann et al. (2004) [46] HOPE study	Vitamin E, 400 IU once daily	993 patients with mild-to-moderate renal insufficiency at high risk for cardiovascular events	No apparent effect on cardiovascular outcomes

*Acetylcysteine *			
Tepel et al. (2003) [47]	Acetylcysteine, 600 mg twice daily	134 HD patients followed for 2 years	(1) Cardiac events reduced by 30% (2) Ischemic stroke reduced by 36%

*Vitamin C *			
Tarng et al. (2004) [48]	Vitamin C, 300 mg three times weekly for 8 weeks	60 HD patients	Mean 8-OH-dG levels decreased significantly in all subjects

*Losartan *			
Kayabasi et al. (2013) [49]	Losartan 50–100 mg once daily	52 HD patients followed for 3 months	Decreasing oxidative stress index and increasing plasma thiol groups

*Folic acid *			
Delfino et al. (2007) [50]	Folic acid, 10 mg three times weekly for 6 months	46 HD patients	Effectively lowered plasma Hcy levels

*Bardoxolone methyl *			
Pergola et al. (2011) [51] BEAM study	Bardoxolone methyl at a target dose of 25, 75, or 150 mg once daily	Adults with CKD	Improved estimated glomerular filtration rate at 24 weeks
